# An Optical Method for Serum Calcium and Phosphorus Level Assessment during Hemodialysis

**DOI:** 10.3390/toxins7030719

**Published:** 2015-02-27

**Authors:** Jana Holmar, Fredrik Uhlin, Anders Fernström, Merike Luman, Joachim Jankowski, Ivo Fridolin

**Affiliations:** 1 Department of Biomedical Engineering, Technomedicum, Tallinn University of Technology, Ehitajate tee 5, 19086 Tallinn, Estonia; E-Mails: fredrik.uhlin@regionostergotland.se (F.U.); merike.luman@regionaalhaigla.ee (M.L.); ivo@cb.ttu.ee (I.F.); 2 Department of Nephrology, Heart- and Medicine Center, Region Östergötland and Department of Medicine and Health Sciences, Linköping University, 581 85 Linköping, Sweden; E-Mail: anders.fernstrom@regionostergotland.se; 3 Centre of Nephrology, North Estonian Medical Centre, Sütiste tee 19, 13419 Tallinn, Estonia; 4 Institute for Molecular Cardiovascular Research, RWTH Aachen University, University Hospital, Pauwelsstraße 30, D-52074 Aachen, Germany; E-Mail: jjankowski@ukaachen.de

**Keywords:** dialysis, vascular calcification, calcium, phosphorus, optical monitoring, ultraviolet absorbance, fluorescence

## Abstract

Survival among hemodialysis patients is disturbingly low, partly because vascular calcification (VC) and cardiovascular disease are highly prevalent. Elevated serum phosphorus (P) and calcium (Ca) levels play an essential role in the formation of VC events. The purpose of the current study was to reveal optical monitoring possibilities of serum P and Ca values during dialysis. Twenty-eight patients from Tallinn (Estonia) and Linköping (Sweden) were included in the study. The serum levels of Ca and P on the basis of optical information, *i.e.*, absorbance and fluorescence of the spent dialysate (optical method) were assessed. Obtained levels were compared in means and SD. The mean serum level of Ca was 2.54 ± 0.21 and 2.53 ± 0.19 mmol/L; P levels varied between 1.08 ± 0.51 and 1.08 ± 0.48 mmol/L, measured in the laboratory and estimated by the optical method respectively. The levels achieved were not significantly different (*p* = 0.5). The Bland-Altman 95% limits of agreement between the two methods varied from −0.19 to 0.19 for Ca and from −0.37 to 0.37 in the case of P. In conclusion, optical monitoring of the spent dialysate for assessing the serum levels of Ca and P during dialysis seems to be feasible and could offer valuable and continuous information to medical staff.

## 1. Introduction

Mortality among end-stage renal disease (ESRD) patients is continuously very high. Only 52% of hemodialysis (HD) and 61% of peritoneal dialysis patients live for at least three years after the beginning of renal replacement therapy. One leading cause of death is cardiovascular disease (CVD) [1]. Vascular calcification is common in chronic kidney disease (CKD) patients, and one of the reasons is abnormal bone metabolism, resulting in high serum levels of calcium (Ca) and phosphorus (P) [2]. It has been shown that elevated phosphorus levels are independently associated with mortality in dialysis patients [3]. The Dialysis Outcomes and Practice Patterns Study (DOPPS) indicated that increased levels of serum Ca, Pa and PTH are related to increased all-cause and cardiovascular mortality [4]. 

Kidney Disease Improving Global Outcomes (KDIGO) and Chronic Kidney Disease–Mineral and Bone Disorder (CKD-MBD) guidelines [5], endorsed by European Renal Best Practice (ERBP) work group [6], suggest that elevated phosphorus levels in ERSD patients should be lowered towards normal range, and serum calcium should be maintained in the normal range. In healthy subjects, serum calcium is usually in the range of 8.5–10.5 mg/dL (2.1–2.6 mmol/L) and serum level of phosphorus between 2.5 and 4.5 mg/dL (0.81–1.45 mmol/L) [5].

Calcium-phosphorus product (Ca × P) can be used for estimation of arterial calcification and cardiovascular event risk in ERSD patients [7,8]. However, use of Ca × P in clinical practice is under debate and a combined evaluation of individual values of serum calcium and phosphorus in favor of estimation of Ca × P is suggested [5].

Efficient control of Ca and P levels in dialysis patients may help to prevent vascular calcification. Further, it has been shown that removal profile of phosphorus during dialysis is different from urea [9]. It is possible to observe effectiveness of removal by analyzing the blood (pre- and post-dialysis) samples, but an alternative option is optical monitoring of the spent dialysate. Methods for optical estimation of dialysis quality [10] and small [11,12], protein bound [13] and middle [14] molecular weight uremic retention solutes removal during dialysis has been proposed. Possibility for optical determination of phosphate elimination during dialysis has been also demonstrated [15]. 

The aim of this study was to explore whether multi-wavelength algorithms based on optical properties, ultraviolet-absorbance (UV) and fluorescence (F) of the spent dialysate are an appropriate technique for estimating serum levels of Ca and P in CKD patients.

## 2. Results

**Figure 1:**
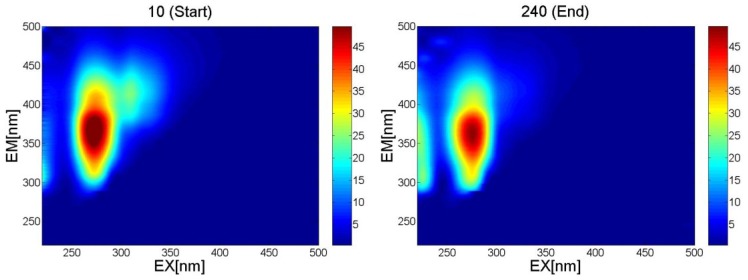
Examples of fluorescence spectra of spent dialysate taken at the start (10 min) and at the end (240 min) of the dialysis procedure. Excitation (EX) = 220–500 nm, Emission (EM) = 220–500 nm.

Fluorescence analysis was performed over an excitation (EX) wavelength range of 220–500 nm (excitation increment 10 nm); emission (EM) spectra over a wavelength range of 220–500 nm were recorded at each excitation and the resulting 3D fluorescence maps are presented in Figure 1. The amplitude of the spectra is proportional to the content of eliminated uremic retention solutes in the spent dialysate.

**Figure 2:**
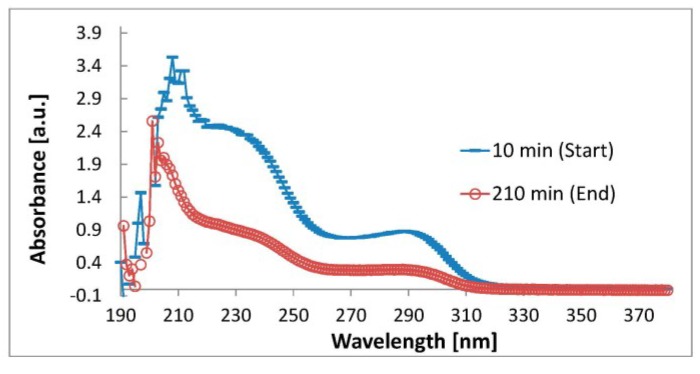
Examples of absorbance spectra of spent dialysate samples taken at the start and at the end of the dialysis over a wavelength range 190–380 nm.

Ultraviolet (UV)-absorbance measurements of the spent dialysate samples were performed over a wavelength range of 190–380 nm (Figure 2). 

**Table 1:** Serum total calcium and phosphorous concentration values estimated by different methods.

	Min	Max	Mean	SD	N	R	R^2^	St. Error of Estimate
**Ca_Lab (mmol/L)**	2.15	2.95	2.54	0.21	83			
**Ca_Opt (mmol/L)**	2.22	2.95	2.53	0.19	83	0.90	0.81	0.093
**P_Lab (mmol/L)**	0.42	2.85	1.08	0.51	142			
**P_Opt (mmol/L)**	0.38	2.76	1.08	0.48	142	0.93	0.87	0.182

Values from the laboratory and estimated by the optical method were compared using paired Student *t*-test; the values are not statistically different (*p* = 0.5).

Obtained models used 6–8 independent variables (UV-absorbance and fluorescence values at certain wavelengths) to estimate the substance level. The values of Ca and P concentrations in serum measured in the laboratory (Ca_Lab, P_Lab) and estimated by the models (Ca_Opt, P_Opt) are presented in Table 1. Serum Ca level was above the standard range in the case of two patients. Serum P level exceeded normal value at the beginning of at least one studied session in the case of 15 patients. 

**Figure 3:**
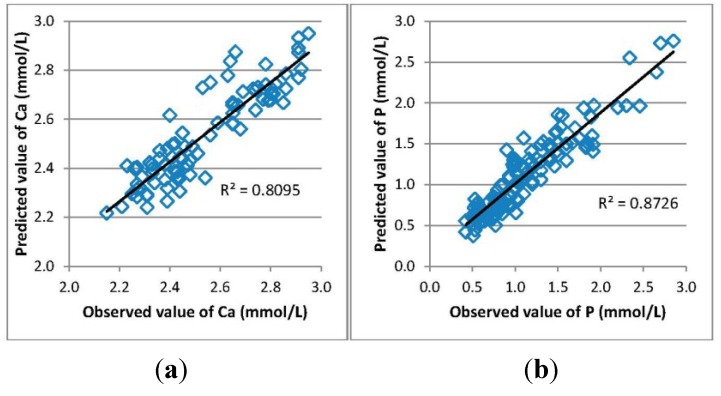
Goodness of fit of the multiple linear regression models for estimating the serum concentration of (**a**) Ca and (**b**) P.

Measured and calculated values of Ca and P in serum samples taken before and after the dialysis procedure were compared and the goodness of fit is shown in Figure 3.

**Figure 4:**
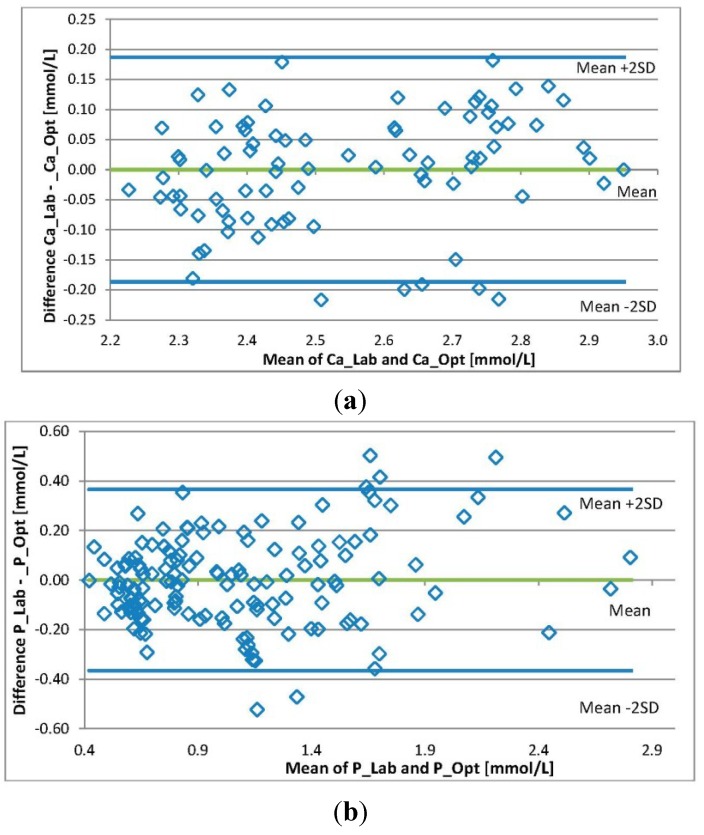
Bland-Altman plots. The individual differences between the levels of (**a**) Ca and (**b**) P concentrations from the laboratory and those estimated by the optical method plotted against the mean value of levels from the lab and the optical method.

The differences in the individual values of a substance level from the laboratory and those calculated by the models are presented in Figure 4. The Bland-Altman analysis shows that the 95% limits of agreement between the two methods varied from −0.19 to 0.19 for Ca and from −0.37 to 0.37 for P.

## 3. Discussion

The primary aim was to develop multi-wavelength algorithms based on the optical information of the spent dialysate for optical estimation of serum level trends of Ca and P. 

It has been suggested that the efficiency of phosphate removal should be monitored in addition to traditional dialysis dose assessment techniques [16] and during HDF procedures phosphate elimination is often more efficient compared to HD procedures [17]. In order to maintain patients’ Ca levels within the desired limits, dialysate concentration of Ca should be individually adjusted according to plasma Ca levels [18]. Further, it has been suggested to use a dialysate consisting Ca between 1.25 and 1.50 mmol/L [4]. Content of Ca in the dialysate solution is the reason for the mean Ca concentration change in spent dialysate samples collected during dialysis sessions is ~5%. At the same time, the P content in the spent dialysate changes 50% on an average. Management of serum P levels in patients with chronic kidney disease is extremely critical to prevent vascular calcification and CVD [19]. Table 1 shows the variability of levels of Ca and P estimated in the course of the study. 

Optical monitoring of hemodialysis has been considered as a beneficial and cost-effective alternative to traditional methods to assess the effectiveness of the dialysis procedure without the need of frequent blood sampling [20]. The optical monitoring possibility of the phosphate removal during dialysis has been suggested by Enberg *et al.* [15]. This study included 11 patients and 33 dialysis sessions, while absorbance at a single wavelength was used. 

Our study involved 28 patients from Tallinn and Linköping. Several of fluorescence and absorbance maxima/minimum of the spent dialysate are shown in Figure 1 and Figure 2. The amplitude of the spectra is proportional to the content of eliminated uremic retention solutes in the spent dialysate, being higher/lower at the beginning of the dialysis treatment (10 min) and lower/higher at the end of the dialysis in particular wavelength regions. Good correlations between the serum levels of Ca and P and the optical parameters of the spent dialysate transformed to serum values (Figure 3) indicate that the serum levels and removal of these substances can be observed optically.

Since Ca and P do not absorb light in the wavelength region measured, it is most likely that the obtained good correlation is induced by surrogate marker.

However, these surrogate markers seem to well express the serum concentration changes of Ca and P. Additionally, the long-term effect on the correlation should be investigated in future research. Obtained levels were not statistically different from the values measured in clinical chemistry laboratories (*p* < 0.05). Although the use of the calcium-phosphorus product (Ca × P) in clinical practice is under debate, it would be possible to perform such (or similar) combined measurements optically. 

Figure 4 illustrates the differences between individual values of the levels of Ca and P. The Bland-Altman analysis shows that the 95% limits of agreement between the two methods varied from −0.19 to 0.19 for Ca and from −0.37 to 0.37 in the case of P. The agreement achieved could be considered adequate for Ca while in the case of P, the limit of agreement is rather wide. An explanation for this may be the large magnitude of the measurement results. However, the method is probably suitable for long-term monitoring of P trends or removal. Performed study demonstrates that it is possible to assess serum levels of Ca and P using optical information of the spent dialysate. In this way, a patient’s serum level of calcification markers can be followed continuously, which may have a beneficial effect on prevention and treatment. 

## 4. Materials and Methods 

The study included 28 chronic hemodialysis patients, 19 males and nine females, mean age 64 ± 13 years. Twenty patients were observed in Tallinn, Estonia and eight patients in Linköping, Sweden. Clinical data of the studied patients is presented in Table 2. The patients were followed in 52 hemodiafiltration (HDF) and 36 hemodialysis (HD) procedures. The studies were performed after authorization of the protocol by the Tallinn Medical Research Ethics Committee, Estonia and Regional Ethical Review Board, Linköping, Sweden. All participating patients gave an informed consent. 

**Table 2:** Data of the studied patients.

Patient	Kidney Disease	Age	Gender	BMI	Time on HD (months)	Dialyzer Type and Membrane Area	Dialysis Access	Procedure Type (HD/HDF)
1	Polycystic kidney disease	83	M	23.5	109	FX80-1.8 m^2^	a/v fistula	HD and HDF
2	Atherosclerosis	57	M	22.5	32	FX80-1.8 m^2^	a/v fistula	HD and HDF
3	Diabetic nephropathy	85	M	18.7	36	FX80-1.8 m^2^	a/v fistula	HD and HDF
4	Hypertensive renal disease	81	F	34.6	12	FX80-1.8 m^2^	graft	HD and HDF
5	Diabetic nephropathy	84	M	24.4	29	FX80-1.8 m^2^	a/v fistula	HD and HDF
6	Polycystic kidney disease	49	M	16.7	45	FX80-1.8 m^2^	a/v fistula	HD and HDF
7	Diabetic nephropathy	76	M	29.0	32	FX80-1.8 m^2^	a/v fistula	HD and HDF
8	Atherosclerosis	73	M	33.2	24	FX80-1.8 m^2^	a/v fistula	HD and HDF
9	Glomerulo-nephritis	69	F	27.1	47	FX10-1.8 m^2^	a/v fistula	HD
10	Tubulointerstitial nephritis	62	F	20.3	7	FX10-1.8 m^2^	a/v fistula	HD
11	Diabetic nephropathy	60	F	26.4	24	FX80-1.8 m^2^	graft	HDF
12	Polycystic kidney disease	68	M	27.8	96	FX80-1.8 m^2^	graft	HDF
13	Polycystic kidney disease	43	M	23.9	16	FX80-1.8 m^2^	graft	HDF
14	Tubulointerstitial nephritis	60	M	27.1	26	FX80-1.8 m^2^	a/v fistula	HDF
15	Hypertensive renal disease	48	M	27.8	8	FX10-1.8 m^2^	a/v fistula	HD
16	Hypertensive renal disease	59	M	26.9	28	FX80-1.8 m^2^	a/v fistula	HDF
17	Myeloma	56	F	30.5	28	FX80-1.8 m^2^	graft	HDF
18	Myeloma	58	M	25.6	29	FX80-1.8 m^2^	a/v fistula	HDF
19	Diabetic nephropathy	74	F	27.0	37	FX10-1.8 m^2^	a/v fistula	HD
20	Polycystic kidney disease	46	M	22.6	42	FX80-1.8 m^2^	graft	HDF
21	Myeloma	58	F	30.5	53	FX100-1.8 m^2^	graft	HDF
22	Tubulointerstitial nephritis	43	F	19.0	23	FX80-1.8 m^2^	a/v fistula	HDF
23	Hypertensive renal disease	73	M	27.1	44	FX80-1.8 m^2^	permanent dialysis catheter	HDF
24	Tubulointerstitial nephritis	56	M	23.9	52	FX80-1.8 m^2^	a/v fistula	HDF
25	Diabetic nephropathy	58	M	37.6	36	FX100-1.8 m^2^	a/v fistula	HDF
26	Hypertensive renal disease	80	M	20.3	81	FX10-1.8 m^2^	a/v fistula	HD
27	Myeloma	60	M	25.3	55	FX100-1.8 m^2^	a/v fistula	HDF
28	Tubulointerstitial nephritis	64	F	23.0	28	FX80-1.8 m^2^	a/v fistula	HD

Fresenius 5008 (Fresenius Medical Care, Bad Homburg, Germany) dialysis machine was used. The dialyzers used were FX10, FX80, FX100 and FX800 (Fresenius Medical Care, Bad Homburg, Germany). Conventional acetic acid acidified solution was used. The treatment durations varied between 180 and 270 min; the dialysate flow was 500 mL/min and the blood flow varied between 250 and 350 mL/min.

The concentrations of total Ca and P in serum samples taken before and after the dialysis procedure were determined in the Clinical Chemistry Laboratories using standardized methods. The Ca method is based on the research of Michaylova and Illkova who discovered that Arsenazo III and Ca could form a stable complex at low pH [21]. The P method originates from the work of Daly and Ertinghausen who described a UV-absorbing complex formation between P and molybdate [22]. Ca concentration was not determined in all procedures. Therefore, the number of Ca results is markedly lower than the number of P results. Serum Ca and P levels at the beginning of dialysis sessions were compared with normal ranges. Spent dialysate samples were collected from the drain tube of the dialysis machine 10 min after the start of the session and at the end of the session. 

Fluorescence analysis was performed over an excitation (EX) wavelength range of 220–500 nm, excitation increment 10 nm was chosen. Emission (EM) spectra over a wavelength range of 220–500 nm were recorded at each excitation. Spectrofluorophotometer (SHIMADZU RF-5301, Kyoto, Japan) was used for the measurements.

UV-absorbance measurements of the spent dialysate samples were performed over a wavelength range of 190–380 nm (Figure 2). UV-VIS-NIR spectrophotometer (V-570, JASCO Corp., Tokyo, Japan) was used in Sweden and UV-3600, SHIMADZU, Japan was used in Tallinn. The wavelengths were chosen on the basis of earlier spent dialysate measurements [23,24].

Measurements were performed at the room temperature (*ca.* 22 °C). The obtained spectral values were processed and presented by software Panorama fluorescence and UV Probe (SHIMADZU, Kyoto, Japan), the final data processing was performed in Excel (Microsoft Office Excel 2007). 

Some of the measured values (concentration/absorbance/fluorescence) were excluded from the data. The criteria for exclusion were incorrect or illogical values of measured concentration or absorption/fluorescence if, for example, sampling was performed simultaneously with self-tests of the dialysis machine when the concentration and the absorbance dropped close to zero.

Models for serum levels of Ca and P calculation were created utilizing information from the measured fluorescence and UV-absorbance. Analysis was performed with Statistica 9.0 (Statsoft Inc., Tulsa, OK, USA) and multiple linear regression (forward stepwise regression method) was employed. The serum level of a substance was set as a dependent parameter, and optical characteristics (UV- absorbance and fluorescence) of the spent dialysate were set as independent parameters. 

The algorithms obtained for the levels (Y) calculation are in the form

*Y* = *a* + *b*_1_*x*_1_ + *b*_2_*x*_2_ +…+ *b_i_x_i_* (1)

where *a* is the intercept, *b* is the slope and *x* is the independent variable (the value of UV-absorbance (A) or fluorescence (F) at a particular wavelength).

*Ca* = *a* + *b*_1_*F*_1_ + *b*_2_*A*_1_ + *b*_3_*A*_2_ + *b*_4_*F*_2_ + *b*_5_*F*_3_ + *b*_6_*A*_3_ (2)

*P* = *a* + *b*_1_*F*_1_ + *b*_2_*F*_2_ + *b*_3_*F*_3_ + *b*_4_*F*_4_ + *b*_5_*A*_1_ + *b*_6_*A*_2_ + *b*_7_*A*_3_ + *b*_8_*A*_4_ (3)

To determine differences between level values from the clinical laboratory and the optical method, the paired two sample for means Student *t*-test (*p* < 0.05 was considered significant) was used. To reveal the level of agreement between the two methods, a Bland-Altman analysis was performed.
